# Knowledge, Attitudes, and Perceptions of Midwives and Obstetricians/Gynecologists on Sexual Activity During Pregnancy: A Cross-Sectional Study in Greece

**DOI:** 10.3390/clinpract16070130

**Published:** 2026-07-10

**Authors:** Eleni Charitopoulou, Angeliki Bolou, Vikentia Harizopoulou, Antigoni Sarantaki, Athina Diamanti, Irene Athina Avramidou, Giannoula Kyrkou

**Affiliations:** 1 Department of Midwifery, School of Health and Care Sciences, University of West Attica, 12243 Athens, Greece; eleni.charitopoulou.mdw@gmail.com (E.C.); abolou@uniwa.gr (A.B.); vharizopoulou@uniwa.gr (V.H.); esarantaki@uniwa.gr (A.S.); adiamanti@uniwa.gr (A.D.); 2 Department of Midwifery, School of Health Sciences, International Hellenic University, 57400 Thessaloniki, Greece; ireneavramidou@gmail.com

**Keywords:** sexual health, pregnancy, midwives, obstetricians/gynecologists, sexual counseling, maternity care

## Abstract

**Background:** Sexual health is a key component of quality of life and an essential aspect of holistic maternity care. During pregnancy, changes in sexual function are often accompanied by fears and misconceptions, which are influenced by healthcare professionals’ counseling. **Objectives**: The present study aimed to investigate the knowledge, attitudes, and perceptions of midwives and obstetricians/gynecologists in Greece regarding sexual activity during pregnancy. **Methods**: A cross-sectional study was conducted using an anonymous, self-administered questionnaire. Participants included 121 healthcare professionals (73 midwives and 48 obstetricians/gynecologists). Data were analyzed using appropriate non-parametric statistical tests and multiple linear regression analysis. **Results**: The majority of participants (97.5%) considered sexual activity during pregnancy to be beneficial. Midwives demonstrated significantly higher scores on the knowledge assessment compared to obstetricians/gynecologists (67.7 ± 18.0 vs. 54.8 ± 24.2, *p* = 0.001) and more frequently selected the questionnaire response indicating benefits for both the couple and the fetus (46.6% vs. 27.1%, *p* = 0.031). A higher proportion of midwives perceived that pregnant women do not receive adequate information regarding their sexual health (93.2% vs. 68.8%, *p* < 0.001). Obstetricians/gynecologists were more likely to recommend restrictions even in low-risk pregnancies. **Conclusions**: The findings highlight the need for improved education and standardized counseling protocols on sexual health during pregnancy, with particular emphasis on strengthening the role of midwives in delivering holistic, woman-centered care.

## 1. Introduction

Sexual health is a fundamental component of overall health and quality of life [1]. During pregnancy, physiological, hormonal, and psychosocial changes can significantly influence women’s sexual function and couples’ intimacy [2,3]. Although international literature indicates that sexual activity is safe in most uncomplicated pregnancies [4], misconceptions, fears, and unnecessary restrictions remain common.

Obstetricians/gynecologists and midwives play a pivotal role in providing evidence-based information and shaping pregnant women’s perceptions regarding sexual activity during gestation. Midwives, in particular, are central to the provision of holistic, woman-centered care, including sexual health counseling. However, previous studies indicate that the knowledge and practices of healthcare professionals vary considerably and are influenced by education, clinical experience, and personal attitudes [5,6].

Limited communication and inadequate counseling on sexual health during pregnancy have been consistently reported in the literature, contributing to persistent myths and unmet informational needs among pregnant women [7,8,9]. These gaps may negatively affect women’s psychological well-being and relationship satisfaction during pregnancy [7,10].

In Greece, maternity care is predominantly physician-led, with midwives often having a less prominent role in antenatal counseling [11,12]. At the same time, there is a lack of systematic evidence examining healthcare professionals’ approaches to sexual health during pregnancy, particularly regarding differences between midwives and obstetricians/gynecologists.

Therefore, this study aimed to investigate the knowledge, attitudes, and perceptions of midwives and obstetricians/gynecologists in Greece regarding sexual activity during pregnancy, with a focus on identifying knowledge gaps, variations in clinical practice, and areas for improvement in sexual health counseling.

### Objectives

Specifically, this study sought to evaluate healthcare professionals’ knowledge of the medical aspects of sexual activity during pregnancy, explore their attitudes and perceptions toward this topic, and examine current counseling practices in clinical care. Furthermore, the study aimed to identify barriers to effective communication and counseling, as well as to assess the educational needs of midwives and obstetricians/gynecologists in order to inform improvements in clinical practice, enhance sexual health counseling, and support evidence-based maternity care.

## 2. Materials and Methods

### 2.1. Study Design

A cross-sectional study design was employed using a convenience sample of healthcare professionals. The study was conducted using an anonymous, self-administered electronic questionnaire, which was distributed to obstetricians/gynecologists and midwives through their respective professional associations. Participants were recruited electronically on a voluntary basis. Because the questionnaire was disseminated through professional associations and open electronic distribution channels, the research team did not have access to the exact number of healthcare professionals who received, viewed, or opened the survey invitation. Therefore, a response rate could not be calculated. Prior to completing the questionnaire, participants were provided with detailed information about the study purpose, confidentiality, and their rights. Electronic informed consent was obtained before participation. The online survey platform was configured to require responses to all questionnaire items before submission; therefore, no missing data were recorded in the final dataset. Data collection took place between April and July 2025. The questionnaire was developed and distributed via an online survey platform (Microsoft Forms).

### 2.2. Eligibility Criteria

This cross-sectional study included obstetricians/gynecologists and midwives actively practicing in Greece, in either the public or private health sector.

#### 2.2.1. Inclusion Criteria

- Obstetricians/gynecologists or midwives with active professional practice in Greece.
- Holding a valid professional license.
- Adequate proficiency in the Greek language to complete the questionnaire.
- Provision of informed consent.

#### 2.2.2. Exclusion Criteria

- Individuals without complete professional qualifications or without a valid license to practice.
- Individuals not actively practicing at the time of the study.
- Failure to provide informed consent.

### 2.3. Instrumentation

The data collection tool was an anonymous, self-administered questionnaire developed by the research team based on the study objectives and a review of the relevant literature. The questionnaire included sections on demographic and professional characteristics, educational background, clinical experience, knowledge, and attitudes and perceptions regarding sexual activity during pregnancy.

The questionnaire was reviewed by experts in midwifery and obstetrics for content validity, and appropriate modifications were made. Content assessment was performed through a multi-step process including literature review, expert review, and pilot testing. Questionnaire items were developed based on evidence identified in the international literature regarding sexual activity during pregnancy and were subsequently evaluated by experts in midwifery and obstetrics for relevance, clarity, and comprehensiveness.

Subsequently, a pilot study was conducted with 10 healthcare professionals to assess clarity, comprehensibility, and feasibility, leading to further minor revisions. The pilot participants were not included in the final sample. No major changes were required following the pilot testing.

Although the instrument was not formally validated, these procedures supported its content relevance and applicability. The questionnaire consisted of thirty-nine (39) items.

### 2.4. Ethics and Institutional Approval

The study was conducted in accordance with the principles of the Declaration of Helsinki. The research protocol was approved by the Ethics and Deontological Committee of the University of West Attica (Protocol Number: 37824/15-04-2025). All participants were fully informed about the purpose of the research and provided their informed consent electronically prior to completing the questionnaire. Participation was anonymous, and the data were collected and analyzed with full respect for personal data protection and confidentiality.

### 2.5. Statistical Analysis

Data were initially tested for normality using the Kolmogorov–Smirnov test. Quantitative variables were expressed as means and standard deviations (SD), while qualitative variables were presented as frequencies and percentages.

A composite knowledge score (0–100%) was calculated from seven questionnaire items assessing knowledge regarding sexual activity during pregnancy. For single-answer questions, correct responses were assigned a value of 1 and incorrect responses a value of 0. For the multiple-response question assessing the definition of sexual activity, a score of 1 was assigned only when all correct response options were selected; otherwise, the response was scored as 0. Each knowledge item contributed equally to the composite score. The total score was converted into a percentage ranging from 0 to 100%, with higher scores indicating greater knowledge regarding sexual activity during pregnancy. Only questionnaire items assessing knowledge contributed to the composite knowledge score; items evaluating attitudes and perceptions were not included.

Comparisons of categorical variables were performed using Pearson’s chi-square test or Fisher’s exact test, as appropriate. Comparisons of the composite knowledge score between two independent groups were performed using the Mann–Whitney U test because the score did not follow a normal distribution.

Multiple linear regression analysis was conducted using a stepwise forward–backward selection method, with the composite knowledge score as the dependent variable. Independent variables included demographic and professional characteristics. Because the composite knowledge score did not follow a normal distribution according to the Kolmogorov–Smirnov test, a logarithmic transformation of the dependent variable was applied prior to regression analysis. The logarithmic transformation was applied to better satisfy the assumptions of linear regression, particularly the normality of model residuals. Regression coefficients (β) and standard errors (SE) were reported.

All *p*-values were two-tailed, and statistical significance was set at *p* < 0.05. Analyses were performed using SPSS software (version 26.0).

## 3. Results

### 3.1. Demographic Characteristics

The study sample consisted of 121 healthcare professionals, the majority of whom were female (76.0%) and aged between 24 and 50 years. Midwives comprised 60.3% of the sample, while 39.7% were obstetricians/gynecologists. Most participants had more than 10 years of professional experience (44.6%) and were primarily practicing in urban areas (87.6%), with Attica and Central Macedonia being the most represented regions. Regarding educational background, over half of the participants held a postgraduate degree; however, only a small proportion (8.3%) had specialized training in sexual health. Detailed demographic characteristics are presented in Table 1.

### 3.2. Attitudes and Perceptions of Healthcare Professionals

The vast majority of participants (97.5%) reported that sexual activity during pregnancy is beneficial. However, most participants (83.5%) indicated that pregnant women do not receive adequate information regarding their sexual health, with midwives reporting this significantly more frequently than obstetricians/gynecologists (93.2% vs. 68.8%, *p* < 0.001). Regarding the provision of counseling, both obstetricians/gynecologists (81.0%) and midwives (74.4%) were identified as appropriate professionals. Notably, midwives were significantly more likely to consider themselves suitable for this role compared to obstetricians/gynecologists (94.5% vs. 43.8%, *p* < 0.001). Significant differences were also observed in clinical decision-making. Obstetricians/gynecologists were more likely to consider a history of infertility or pregnancy following in vitro fertilization as contraindications to sexual activity compared to midwives (*p* = 0.006 and *p* = 0.049, respectively). The aforementioned data are illustrated in Table 2.

### 3.3. Knowledge Levels of Midwives and Obstetricians/Gynecologists

No significant differences were observed between the two groups regarding the perception that sexual activity is generally safe during pregnancy under certain conditions. Similarly, high proportions in both groups recognized the positive impact of sexual activity on maternal psychological well-being. However, midwives were significantly more likely to select the questionnaire response indicating benefits of sexual activity for both the couple and the fetus compared to obstetricians/gynecologists (46.6% vs. 27.1%, *p* = 0.031). Overall questionnaire-based knowledge scores were significantly higher among midwives (67.7 ± 18.0) than among obstetricians/gynecologists (54.8 ± 24.2, *p* = 0.001). Detailed results are presented in Table 3.

**Figure 1:**
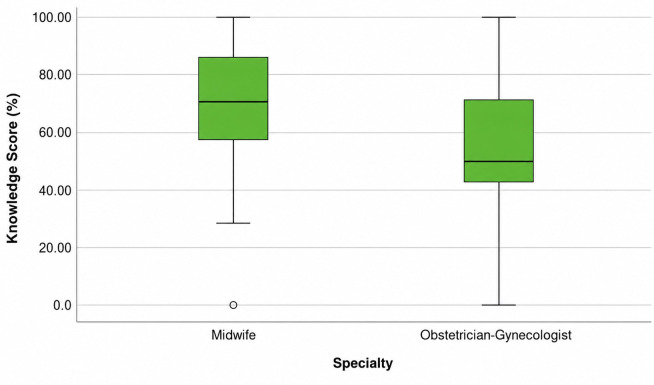
Knowledge test results.

A total knowledge score was calculated as a percentage, assigning one point for each correct response. Midwives demonstrated significantly higher knowledge scores compared to obstetricians/gynecologists (Figure 1).

To identify factors independently associated with knowledge scores, a multiple linear regression analysis was performed, with knowledge score as the dependent variable and demographic and professional characteristics as independent variables (Table 4). A stepwise method was applied. The analysis showed that specialty and participation in sexual health training were significantly associated with knowledge scores. Specifically, obstetricians/gynecologists had lower questionnaire-based knowledge scores than midwives (β = −0.143, *p* = 0.013). In contrast, participation in a training program or seminar on sexual health within the past 5 years was associated with higher questionnaire-based knowledge scores (β = 0.112, *p* = 0.049). Years of professional experience were not significantly associated with questionnaire-based knowledge scores after adjustment.

### 3.4. Clinical Practice

In clinical practice, obstetricians/gynecologists were more likely to recommend moderated sexual activity, whereas midwives more frequently advised that sexual activity could be continued without restrictions in low-risk pregnancies. Most participants reported feeling comfortable discussing sexual health issues with pregnant women (72.7%), with midwives reporting higher levels of comfort than obstetricians/gynecologists. A statistically significant association was found between prior training in sexual health and increased comfort in discussing these issues (*p* < 0.001). Detailed results are presented in Table 5.

Furthermore, a significant association was observed between healthcare professionals’ understanding of sexual activity and their clinical counseling practices (Table 6). Participants who correctly defined sexual activity were significantly more likely to recognize that there are no gestational week restrictions for sexual intercourse in uncomplicated pregnancies (*p* = 0.037 and *p* = 0.049). In addition, those with a correct definition of sexual activity were more likely to recommend that pregnant women without risk factors could engage in sexual activity without restrictions, whereas those with an incorrect definition more frequently advised moderation or restrictions (*p* = 0.002). These findings suggest a potential association between knowledge and clinical counseling practices.

The most commonly reported barriers to discussing sexual health were limited consultation time (48.8%) and lack of specialized training (27.3%), with no significant differences between professional groups. Less experienced professionals more frequently reported lack of knowledge as a barrier, while those working in urban areas more often identified time constraints (Table 7).

## 4. Discussion

This study highlights important gaps in healthcare professionals’ knowledge, attitudes, and clinical practices regarding sexual activity during pregnancy, while also revealing significant differences between midwives and obstetricians/gynecologists.

The demographic characteristics of the sample provide important context for interpreting these findings. The inclusion of healthcare professionals from multiple geographic regions across Greece enhances the representativeness of the study, although the predominance of participants working in urban areas should be considered when generalizing the results. The higher participation of midwives may reflect greater engagement with issues related to sexual health and counseling during pregnancy, while also suggesting differences in professional priorities or interest in the study topic.

Despite the relatively high level of academic qualifications among participants, only a small proportion had received specialized education in sexual health, and most had not participated in relevant training programs in recent years. This gap between general academic education and targeted training may contribute to the deficiencies observed in both knowledge and counseling practices. Notably, years of professional experience were not associated with improved knowledge, indicating that clinical experience alone is insufficient without continuous professional development, as also reported in previous studies [13].

The findings further reveal that sexual health is not consistently addressed during pregnancy, as a large proportion of participants reported that women do not receive adequate information. This is consistent with previous research indicating that healthcare professionals do not routinely provide structured counseling on sexual activity, leading to gaps in patient knowledge and reliance on informal sources of information [14]. Recent evidence suggests that structured psychological and counseling interventions may improve sexual satisfaction during pregnancy, further highlighting the importance of addressing sexual health within routine maternity care [15]. These findings should also be considered within the broader organization of prenatal care services. Evidence from European publicly funded prenatal care systems suggests that the organization, accessibility, and continuity of antenatal care influence the delivery of evidence-based counseling and patient education. Strengthening counseling on sexual health should therefore be viewed not only as an individual professional responsibility but also as an integral component of comprehensive prenatal care services [16].

Differences in professional roles were also evident, with midwives more likely to consider themselves appropriate providers of sexual health counseling. This aligns with existing literature emphasizing the role of midwives in delivering holistic, woman-centered care and highlights the influence of professional attitudes on clinical practice [17].

In contrast, obstetricians/gynecologists appeared more likely to adopt restrictive approaches, particularly in cases such as infertility or assisted reproduction, despite the general safety of sexual activity in uncomplicated pregnancies. This tendency may reflect a more cautious, risk-oriented approach influenced by medical training and has also been described in previous research [2].

Although general awareness of the safety and benefits of sexual activity during pregnancy was observed, important knowledge gaps and misconceptions persist, particularly regarding fetal safety and appropriate counseling practices [2]. It should be noted, however, that current evidence primarily supports the safety of sexual activity in uncomplicated pregnancies and its potential benefits for maternal well-being and couple relationships. Any potential positive influence on pregnancy outcomes or fetal well-being is considered indirect and is likely mediated through improved maternal physical and psychological well-being, rather than through a direct effect of sexual activity on fetal development. Direct beneficial effects on fetal development remain insufficiently established and should therefore be interpreted with caution. In addition, variability in the definition of sexual activity, particularly regarding non-penetrative behaviors, suggests inconsistencies in conceptual understanding that may influence both counseling practices and patient perceptions [14].

Midwives demonstrated higher knowledge scores compared to obstetricians/gynecologists, a finding that may reflect differences in training focus and professional roles in maternity care [17]. However, alternative explanations should also be considered, including differences in educational background, professional interest in sexual health, and potential self-selection bias, as healthcare professionals with greater interest in the topic may have been more likely to participate in the study. Importantly, participation in sexual health training was independently associated with higher questionnaire-based knowledge levels, suggesting a potential role of targeted education in strengthening professional competence [17]. In contrast, years of professional experience were not associated with knowledge levels, underscoring the importance of continuous education rather than reliance on clinical exposure alone [18].

A key finding of this study is the clear association between questionnaire-based knowledge and clinical practice. Healthcare professionals with a more accurate understanding of sexual activity were more likely to provide evidence-based counseling, including the avoidance of unnecessary restrictions. Conversely, limited or incorrect knowledge was associated with more cautious or restrictive recommendations, even in the absence of clinical indications. These findings indicate that questionnaire-based knowledge levels may be associated with clinical counseling practices and highlight the importance of addressing misconceptions through education.

In terms of clinical practice, most healthcare professionals reported feeling comfortable discussing sexual health with pregnant women, particularly midwives. However, a notable proportion still reported avoiding the initiation of such discussions, suggesting that perceived comfort may not always translate into clinical behavior. Previous research has identified similar discrepancies, often attributed to a lack of training, uncertainty, or discomfort in addressing sensitive topics [19].

Pregnant women were generally reported to respond positively when discussions on sexual health were initiated, suggesting that barriers may originate more from healthcare professionals than from patients. This finding aligns with previous research indicating that women are receptive to discussing sexual health when given the opportunity [20]. However, patient- and partner-related factors, such as negative attitudes or reluctance to engage in discussion, were also identified, highlighting the influence of sociocultural factors on communication [19].

The absence of significant differences between professional groups in reported barriers suggests that these challenges are systemic rather than profession-specific. Therefore, addressing these barriers requires both targeted educational interventions and structural changes within clinical settings to facilitate open and effective communication on sexual health during pregnancy.

### Strengths and Limitations

The cross-sectional design of this study does not allow causal inferences. Additionally, the use of convenience sampling and voluntary participation may have introduced selection bias, while the higher participation of midwives may have influenced comparisons between professional groups. The reliance on self-reported data may also be subject to social desirability bias. Although the questionnaire was not formally validated, it was developed based on international literature, underwent expert review, and was pilot-tested. Furthermore, the predominance of participants from urban areas and the higher representation of midwives may limit the representativeness of the sample and should be considered when interpreting the findings and their generalizability. Therefore, the findings should be interpreted as reflecting the characteristics of the participating healthcare professionals and should be generalized with caution to the wider population of midwives and obstetricians/gynecologists in Greece. The regression analysis should be considered exploratory and interpreted with caution.

Despite these limitations, this study has several important strengths. To our knowledge, it is the first study in Greece to directly compare midwives and obstetricians/gynecologists regarding knowledge, attitudes, and clinical practices related to sexual activity during pregnancy. Moreover, it identifies a clear association between knowledge and clinical counseling practices, highlighting the relevance of this association for improving clinical care and guiding targeted professional education.

## 5. Conclusions

This study identified significant differences between midwives and obstetricians/gynecologists in knowledge, attitudes, and clinical practices regarding sexual activity during pregnancy. Although most healthcare professionals recognized the importance of sexual health, important gaps remain, particularly among obstetricians/gynecologists.

Midwives demonstrated higher questionnaire-based knowledge levels and were more likely to support non-restrictive counseling in low-risk pregnancies, highlighting their potential contribution to holistic, woman-centered care. Participation in sexual health education was positively associated with both knowledge and clinical confidence.

These findings support the need for targeted educational interventions and the development of standardized counseling approaches that may enhance sexual health communication and support evidence-based maternity care.

## Figures and Tables

**Table 1:** Demographic Characteristics of the Sample (N = 121).

		N	%
Age	24–30	37	30.6
	31–40	25	20.7
	41–50	33	27.3
	51–60	21	17.4
	>60	5	4.1
Gender	Male	29	24.0
	Female	92	76.0
Married/Civil partnership	Single	43	35.5
	Married or in a Civil Partnership	66	54.5
	Divorced	11	9.1
	Widowed	1	0.8
	Other	0	0.0
Children	No	49	40.5
	Yes	72	59.5
Specialty	Midwife	73	60.3
	Obstetrician/Gynecologist (OB/GYN)	48	39.7
Basic Degree	Midwifery (Greek University)	70	57.9
	Midwifery (International University)	3	2.5
	Medicine (Greek University)	34	28.1
	Medicine (International University)	14	11.6
Master	No	54	44.6
	Yes, related to sexual health	10	8.3
	Yes, unrelated to sexual health	57	47.1
PhD	No	103	85.1
	Yes, related to sexual health	1	0.8
	Yes, unrelated to sexual health	17	14.0
Have you received specialized training in sexual health and sexuality during pregnancy?	No	53	43.8
	Yes, during undergraduate studies	42	34.7
	Yes, through postgraduate training or seminars	26	21.5
Have you participated in any educational program or seminar on sexual health in the past 5 years?	No	85	70.2
	Yes	36	29.8
Type of Working Environment			
Public Hospital		33	27.3
Private Hospital/Clinic		34	28.1
Health Center		10	8.3
Private Practice/Office		57	47.1
University/Research Center		10	8.3
Other		6	5.0
Years of Professional Experience	0–5	41	33.9
	6–10	26	21.5
	11–20	27	22.3
	>20	27	22.3
In which area do you work?	Urban	106	87.6
	Semi-urban	12	9.9
	Rural area	3	2.5
Geographic Region	Attica	38	31.4
	Central Greece	6	5.0
	Central Macedonia	36	29.8
	Thrace	6	5.0
	Western Macedonia	6	5.0
	Epirus	3	2.5
	Thessaly	9	7.4
	Peloponnese	3	2.5
	Ionian Islands	3	2.5
	Aegean Islands	5	4.1
	Crete	6	5.0

**Table 2:** Attitudes and Perceptions of Midwives and Obstetricians/Gynecologists.

	Total Ν (%)	Midwives N (%)	Obstetricians/Gynecologists Ν (%)	*p*
**Do you believe pregnant women receive adequate information?**				
No	101 (83.5)	68 (93.2)	33 (68.8)	**<0.001 ++**
Yes	3 (2.5)	1 (1.4)	2 (4.2)	
I don’t know/Uncertain	17 (14)	4 (5.5)	13 (27.1)	
**Do you believe pregnant women should be encouraged to discuss sexual concerns with healthcare professionals? (multiple answers)**				
Yes, with their gynecologist	98 (81)	55 (75.3)	43 (89.6)	0.060 +
Yes, with their midwife	90 (74.4)	69 (94.5)	21 (43.8)	<0.001 +
Yes, with another healthcare professional (e.g., psychologist)	56 (46.3)	35 (47.9)	21 (43.8)	**0.651 +**
No	3 (2.5)	2 (2.7)	1 (2.1)	>0.999 ++
**What do you consider contraindications for sexual intercourse during pregnancy? (multiple answers)**				
Vaginal bleeding	117 (96.7)	71 (97.3)	46 (95.8)	0.649 ++
Low placental attachment	106 (87.6)	60 (82.2)	46 (95.8)	**0.026 +**
Cervical cerclage	104 (86)	62 (84.9)	42 (87.5)	0.691 +
Premature rupture of membranes	114 (94.2)	67 (91.8)	47 (97.9)	0.242 ++
History of preterm birth	62 (51.2)	37 (50.7)	25 (52.1)	>0.999 ++
History of recurrent miscarriage	43 (35.5)	28 (38.4)	15 (31.3)	0.424 +
History of infertility	8 (6.6)	1 (1.4)	7 (14.6)	**0.006 ++**
Current pregnancy after IVF	20 (16.5)	8 (11)	12 (25)	**0.049 ++**
Current pregnancy after insemination	8 (6.6)	3 (4.1)	5 (10.4)	0.262 ++
Mother’s age >35 years	8 (6.6)	3 (4.1)	5 (10.4)	0.263 ++
Vaginal infections	75 (62)	47 (64.4)	28 (58.3)	0.502 +
Mother’s desire	61 (50.4)	37 (50.7)	24 (50)	0.941 +
Partner’s desire	49 (40.5)	29 (39.7)	20 (41.7)	0.852 +

+ Pearson’s X2 test. ++ Fisher’s exact test. The bold formatting in the tables indicates the statistically significant results (*p*-value).

**Table 3:** Knowledge of sexual activity during pregnancy.

		Total N (%)	Midwives N (%)	Obstetricians/Gynecologists N (%)	*p*
Do you believe it is safe for a pregnant woman to be sexually active? *	Yes	30 (24.8)	15 (20.5)	15 (31.3)	0.103 +
	Yes, under conditions	88 (72.7)	57 (78.1)	31 (64.6)	
	No	2 (1.7)	1 (1.4)	1 (2.1)	
	Don’t know	1 (0.8)	0 (0)	1 (2.1)	
Do you believe sexual activity is beneficial? *	Yes, for the woman	7 (5.8)	1 (1.4)	6 (12.5)	**0.031 +**
	Yes, for the couple	64 (52.9)	37 (50.7)	27 (56.3)	
	Yes, for the couple and the fetus	47 (38.8)	34 (46.6)	13 (27.1)	
	No	2 (1.7)	1 (1.4)	1 (2.1)	
	Don’t know	1 (0.8)	0 (0)	1 (2.1)	
Do you believe sexual activity during pregnancy contributes to the psychological well-being of the pregnant woman? *	No	2 (1.7)	2 (2.7)	0 (0)	0.213 ++
	Yes	115 (95)	71 (97.3)	44 (91.7)	
	Don’t know	4 (3.3)	0 (0)	4 (8.3)	
Do you believe sexual activity affects fetal development? *	No, sex does not affect fetal development	34 (28.1)	17 (23.3)	17 (35.4)	0.070 ++
	Yes, sex positively affects fetal development	46 (38)	32 (43.8)	14 (29.2)	
	Yes, sex negatively affects fetal development	1 (0.8)	1 (1.4)	0 (0)	
	Don’t know	40 (33.1)	23 (31.5)	17 (35.4)	
From which week of pregnancy do you consider it safe for a pregnant woman without complications to have sexual intercourse? *	5th	7 (5.8)	1 (1.4)	6 (12.5)	0.231 +
	8th	4 (3.3)	2 (2.7)	2 (4.2)	
	12th	35 (28.9)	21 (28.8)	14 (29.2)	
	16th	9 (7.4)	5 (6.8)	4 (8.3)	
	20th	2 (1.7)	2 (2.7)	0 (0)	
	24th	1 (0.8)	1 (1.4)	0 (0)	
	28th	1 (0.8)	1 (1.4)	0 (0)	
	32nd	0 (0)	0 (0)	0 (0)	
	36th	4 (3.3)	2 (2.7)	2 (4.2)	
	40th	1 (0.8)	1 (1.4)	0 (0)	
	No restrictions	56 (46.3)	37 (50.7)	19 (39.6)	
	None	1 (0.8)	0 (0)	1 (2.1)	
Up to which week of pregnancy do you consider it safe for a pregnant woman without complications to have sexual intercourse? *	5th	0 (0)	0 (0)	0 (0)	0.173 +
	8th	0 (0)	0 (0)	0 (0)	
	12th	1 (0.8)	0 (0)	1 (2.1)	
	16th	0 (0)	0 (0)	0 (0)	
	20th	0 (0)	0 (0)	0 (0)	
	24th	0 (0)	0 (0)	0 (0)	
	28th	0 (0)	0 (0)	0 (0)	
	32nd	4 (3.3)	4 (5.5)	0 (0)	
	36th	7 (5.8)	2 (2.7)	5 (10.4)	
	40th	9 (7.4)	4 (5.5)	5 (10.4)	
	No restrictions	98 (81)	62 (84.9)	36 (75)	
	None	2 (1.7)	1 (1.4)	1 (2.1)	
How do you define “sexual activity”? (multiple answers) *	Vaginal penetration	116 (95.9)	71 (97.3)	45 (93.8)	
	Orgasm	96 (79.3)	64 (87.7)	32 (66.7)	
	Masturbation	84 (69.4)	55 (75.3)	29 (60.4)	
	Other	3 (2.5)	2 (2.7)	1 (2.1)	0.090 +
**Knowledge score on sexual activity during pregnancy, Mean (SD)**		62.3 (21.5)	67.7 (18.0)	54.8 (24.2)	**0.001 ÷**

+ Pearson’s X2 test. ++ Fisher’s exact test. ÷ Mann–Whitney test. In questions marked with *, the *p*-value refers to the comparison of correct answers. For the other questions, there are no right or wrong answers. The background color indicates the correct answers. The bold formatting in the tables indicates the statistically significant results (*p*-value).

**Table 4:** Factors correlated with the knowledge test.

	β +	SE ++	b ÷	*p*
**Specialty (OB/GYN vs. Midwife)**	−0.143	0.057	−0.223	**0.013**
**Participation in a training program or seminar on sexual health within the last 5 years? (Yes vs. No)**	0.112	0.041	0.182	**0.049**

β +: Unstandardized coefficient, SE ++: Standard Error, b ÷: Standardized coefficient, *p*: *p*-value, Note: A logarithmic transformation of the dependent variable has been applied. The bold formatting in the tables indicates the statistically significant results (*p*-value).

**Table 5:** Issues in daily Clinical Practice.

	Total	Midwives	Obstetricians/Gynecologists	*p*
	**N (%)**	**N (%)**	**N (%)**	
**How comfortable do you feel discussing sexual health matters with pregnant patients?**				
Completely uncomfortable	2 (1.7)	1 (1.4)	1 (2.1)	0.278 ++
Somewhat uncomfortable	5 (4.1)	2 (2.7)	3 (6.3)	
Neutral	7 (5.8)	2 (2.7)	5 (10.4)	
Somewhat comfortable	19 (15.7)	11 (15.1)	8 (16.7)	
Completely comfortable	88 (72.7)	57 (78.1)	31 (64.6)	
Mean (SD) (1: Very uncomfortable–5: Very comfortable)	4.5 (0.9)	4.7 (0.8)	4.1 (1.0)	
**How do pregnant women usually react when you initiate a discussion about their sexual function?**				
I avoid initiating a discussion with the pregnant woman	17 (14)	10 (13.7)	7 (14.6)	**0.009 ++**
Very negatively	0 (0)	0 (0)	0 (0)	
Negatively	4 (3.3)	3 (4.1)	1 (2.1)	
Neutral	36 (29.8)	26 (35.6)	10 (20.8)	
Positively	54 (44.6)	33 (45.2)	21 (43.8)	
Very positively	10 (8.3)	1 (1.4)	9 (18.8)	
**Mean (SD)** (0: I avoid initiating a discussion with the pregnant woman–5: Very positively)	3.2 (1.4)	3.0 (1.3)	3.4 (1.6)	

++ Fisher’s exact test.

**Table 6:** Definition of S.A. and counseling by HCPs.

		Correct Definition of Sexual Activity				*p*
		No		Yes		
		Ν	%	Ν	%	
**No gestational week restriction for sexual intercourse in uncomplicated pregnancies.**	Incorrect	28	66.7	37	46.8	**0.037 +**
	Correct	14	33.3	42	53.2	
**No restriction on the gestational week until which sexual intercourse is safe in uncomplicated pregnancies.**	Incorrect	12	28.6	11	13.9	**0.049 +**
	Correct	30	71.4	68	86.1	
**What advice do you give to a pregnant woman without risk factors when she asks for guidance regarding her sexual activity?**	To have intercourse whenever and as often as she wishes	9	21.4	41	51.9	**0.002 ÷**
	To have intercourse in moderation	12	28.6	8	10.1	
	To use a condom during intercourse	11	26.2	18	22.8	
	To have intercourse, but avoid certain positions	8	19.0	12	15.2	
	Sexual abstinence	2	4.8	0	0.0	

+ Pearson’s X2 test. ÷ Mann–Whitney test. The bold formatting in the tables indicates the statistically significant results (*p*-value). The bold formatting in the tables indicates the statistically significant results (*p*-value).

**Table 7:** Barriers in daily Clinical Practice.

	Total	Midwives	Obstetricians/Gynecologists	*p*
	N (%)	N (%)	N (%)	
**What are the main barriers that prevent you from discussing sexual health issues with the pregnant women under your care?**				
**Lack of available time during the examination/visit**	59 (48.8)	35 (47.9)	24 (50)	0.825 +
**Insufficient specialized education**	33 (27.3)	22 (30.1)	11 (22.9)	0.383 +
**Lack of clinical experience**	17 (14)	11 (15.1)	6 (12.5)	0.691 +
**Insufficient knowledge in managing sexual dysfunction or patient complaints**	25 (20.7)	17 (23.3)	8 (16.7)	0.379 +
**Negative attitude of the pregnant woman**	31 (25.6)	23 (31.5)	8 (16.7)	0.067 +
**Partner’s negative attitude**	30 (24.8)	18 (24.7)	12 (25)	0.966 +
**Other**	7 (5.8)	4 (5.5)	3 (6.3)	0.859 +

+ Pearson’s X2 test.

## Data Availability

The data presented in this study are available on request from the corresponding author.

## Abbreviations

The following abbreviations are used in this manuscript:

UNIWA	University of West Attica
OB/GYN	Obstetrician/Gynecologist
HCPs	Healthcare Professionals
IVF	In Vitro Fertilization
S.A.	Sexual Activity
SD	Standard Deviation
N	Total Number of Participants (Sample Size)
*p*	*p*-value (Probability Value)
SE	Standard Error
β/b	Regression Coefficients (Unstandardized/Standardized)
SPSS	Statistical Package for the Social Sciences
